# Mobile Application and Machine Learning-Driven Scheme for Intelligent Diabetes Progression Analysis and Management Using Multiple Risk Factors

**DOI:** 10.3390/bioengineering11111053

**Published:** 2024-10-22

**Authors:** Huaiyan Jiang, Han Wang, Ting Pan, Yuhang Liu, Peiguang Jing, Yu Liu

**Affiliations:** 1 School of Microelectronics, Tianjin University, Tianjin 300072, China; jianghyan@tju.edu.cn (H.J.); han_wang@tju.edu.cn (H.W.); panting2022@tju.edu.cn (T.P.); liuyuhang@tju.edu.cn (Y.L.); 2 School of Electrical and Information Engineering, Tianjin University, Tianjin 300072, China; pgjing@tju.edu.cn; 3 Zhejiang International Institute for Innovative Design and Intelligent Manufacturing, Tianjin University, Shaoxing 312077, China

**Keywords:** diabetes progression analysis, HbA1c dynamic prediction, mobile application, machine learning, deep learning

## Abstract

Diabetes mellitus is a chronic disease that affects over 500 million people worldwide, necessitating personalized health management programs for effective long-term control. Among the various biomarkers, glycated hemoglobin (HbA1c) is a crucial indicator for monitoring long-term blood glucose levels and assessing diabetes progression. This study introduces an innovative approach to diabetes management by integrating a mobile application and machine learning. We designed and implemented an intelligent application capable of collecting comprehensive data from diabetic patients, creating a novel diabetes dataset named DiabMini with 127 features of 88 instances, including medical information, personal information, and detailed nutrient intake and lifestyle. Leveraging the DiabMini, we focused the analysis on HbA1c dynamics due to their clinical significance in tracking diabetes progression. We developed a stacking model combining eXtreme Gradient Boosting (XGBoost), Support Vector Classifier (SVC), Extra Trees (ET), and K-Nearest Neighbors (KNN) to explore the impact of various influencing factors on HbA1c dynamics, which achieved a classification accuracy of 94.23%. Additionally, we applied SHapley Additive exPlanations (SHAP) to visualize the contributions of risk factors to HbA1c dynamics, thus clarifying the differential impacts of these factors on diabetes progression. In conclusion, this study demonstrates the potential of integrating mobile health applications with machine learning to enhance personalized diabetes management.

## 1. Introduction

Diabetes, a metabolic disorder resulting from insufficient insulin secretion or abnormal cellular response to insulin, is increasingly prevalent. Recent statistics indicate a rise from 536.6 million diabetes cases in 2021 to an estimated 783.2 million by 2045 [1]. Chronic hyperglycemia from diabetes progression leads to severe complications, including cardiovascular dysfunction, kidney disease, retinopathy, stroke, and neuropathy [2]. Diabetes is primarily categorized into type 1, type 2, and gestational diabetes, with type 2 diabetes being the most prevalent, comprising approximately 90% of all diabetes cases [3]. Notably, diabetes is often considered an incurable condition, necessitating lifelong management and careful control once diagnosed.

Diabetes management requires a comprehensive understanding of various factors, including genetic predispositions, environmental influences, lifestyle choices, and dietary content. For example, a BMI of 23 kg/m^2^ or higher is associated with a 43% increased risk of diabetes in men and a 41% increased risk in women, compared to a BMI of 18.5–22.9 kg/m^2^ [4]. Moreover, nutrient intake is significantly linked to diabetes risk [5]. Recent studies found that appropriate vitamin C supplementation can effectively prevent diabetes, whereas iron overload increases diabetes risk [6,7].

In the current medical field, precision medicine is deeply rooted in chronic disease prevention and treatment. As diabetes management models shift from traditional strategies to targeted data-driven precision care, the systematic collection of patient data becomes indispensable. Diabetes research relies on longitudinal, diverse sources and extensive sample data. However, two noteworthy challenges remain in this field. The first challenge is the development for convenient and sustainable data collection methods. Commonly used approaches, like questionnaires and follow-ups, typically involve labor-intensive and resource-demanding procedures and can lead to incomplete or inaccurate data due to recall bias. The second challenge lies in obtaining comprehensive diabetes risk data. Numerous efforts are made to establish diabetes datasets, including Sklean Diabetes Dataset [8], PIMA Indian Diabetes Dataset (PID) [9], and Iraqi Patiant Dataset of Diabetes (IPDD) [10], which contain personal and medical information. Nonetheless, crucial risk factors such as lifestyle and nutrient intake are often underrepresented, and these datasets primarily aim to diagnose diabetes rather than study disease control in diabetic patients.

Given the importance of diabetes management and the proven efficacy of machine learning in the medical field [11,12,13], numerous studies have constructed models to elucidate the intricate relationships between various factors and HbA1c. As a biomarker reflecting long-term blood glucose control over roughly two to three months [14], HbA1c is critical in assessing disease progression. The research employed kernel machine learning and convolutional neural networks for HbA1c estimation using continuous glucose monitoring (CGM) or self-monitored blood glucose data [15,16]. Models such as support vector machine (SVM), random forest (RF), and logistic regression (LR) have been combined with electronic health records to identify individuals at risk of elevated HbA1c [17]. Other studies compared multiple machine learning algorithms like multi-layer perception (MLP) or generalized linear models to classify HbA1c status by clinical data [18,19]. Some research also explored the relationship between retinal fundus photographs, electrocardiography features, and HbA1c levels [20,21]. While these methods have advanced HbA1c management, there remains room for improvement. The data collection process is often complex, continuous sensor usage posing potential infection risks, and focusing on CGM data may overlook vital determinants such as dietary intake and lifestyle.

Considering diabetes is a multifaceted and heterogeneous condition, often incurable and which requires long-term management. Therefore, developing a tool that supports sustained data collection and incorporates various risk factors into an in-depth analysis of disease progression is essential for affected individuals and precision diabetes management. To address these challenges, we developed a solution that leverages a mobile application for data collection and applies appropriate machine learning algorithms for effective data analysis. The integration of these components provides a deeper understanding of the complex factors driving diabetes progression.

As part of this solution, we proposed an artificial intelligence (AI)-enabled mobile system based on smartphones to facilitate comprehensive data collection. This system was implemented as a smartphone application integrating multiple functions, including personal information recording, dietary nutrition tracking, personalized nutritional analysis, and online health and dietary guidance. For personal information collection, the system efficiently gathers basic user data, such as height and weight, as well as lifestyle habits that capture individual characteristics. For dietary nutrition tracking, the system supports three input methods: typing input, voice recognition, and image recognition. To ensure detailed dietary records, we established an extensive food-nutrition database, encompassing 1300 ingredients, over 5200 dishes, and more than 20,000 packaged foods, along with their corresponding nutritional compositions.

Through this dedicated mobile application, we developed a novel diabetes dataset named DiabMini, which integrates personal information, medical information, and detailed lifestyle and nutrient intake data. DiabMini comprises 88 instances, each characterized by 127 features, including gender, weekly exercise, protein intake, body fat percentage (BFP), and HbA1c, alongside other pertinent variables. Building on the DiabMini, we apply machine learning techniques to classify HbA1c changes. We constructed a stacking model tailored to the structure of the DiabMini dataset using the four typical machine learning algorithms, namely SVC, ET, XGBoost, and KNN. This model aims to categorize HbA1c fluctuations based on the demographic, lifestyle, and nutrient features available within the dataset.

The main contributions of this paper are delineated as follows:

- Designed and implemented an AI-enabled mobile system that integrates deep learning techniques to support multiple data collection methods, with a comprehensive system database to facilitate the collection of detailed and diverse patient data.
- Introduced the novel DiabMini dataset, which includes 127 features from 88 diabetic patients, covering personal, medical, and detailed dietary nutrition and lifestyle data. This dataset enables a more holistic and precise analysis of factors affecting diabetes progression.
- Focused on HbA1c as a critical indicator of diabetes progression, we developed a stacking model integrating XGBoost, SVC, ET, and KNN to assess the relationship between various risk factors and HbA1c dynamics, achieving a classification accuracy of 94.23%.
- Applied SHAP to illustrate the contributions of different influencing factors to HbA1c, improving the interpretability of the model’s predictions.
- Supported the advancement of diabetes research by combining continuous and detailed data collection with thorough data analysis, enabling a deeper understanding of diabetes management and progression.

The rest of this paper is organized as follows. Section 2 describes the AI-enabled mobile system, the DiabMini dataset, and the design of classification experiments using HbA1c as a marker of diabetes progression. Section 3 presents the HbA1c classification results and explores the model’s interpretability. Section 4 discusses the main findings, summarizes our work, and outlines future research directions.

## 2. Materials and Methods

This study presents an integrated approach to diabetes management that combines effective data collection techniques with reliable data analysis methods. We propose an AI-enabled mobile system that implements multiple input methods, including typing, voice, and photo, to facilitate dietary recording and evaluation. The system leverages deep learning for voice and photo processing and incorporates an inclusive system database for automatic food recognition and nutritional analysis. To analyze diabetes progression, we developed the DiabMini dataset through the mobile system, which comprises data from 88 participants with 127 features spanning personal, medical, lifestyle, and nutrient intake information. HbA1c changes, a key diabetes progression marker, were categorized into three classes (no change, increased, and decreased). A stacking model combining XGBoost, SVC, ET, and KNN was used to predict HbA1c dynamics. SMOTE oversampling and StratifiedKFold were applied to address the class imbalance and ensure model robustness [22,23]. The following sections will detail the system architecture and data analysis methods. By evaluating the model’s performance, including accuracy, macro-precision, macro-recall, and macro-F1 score, along with the ROC curve and confusion matrix, we will explore how this approach supports the understanding and management of diabetes.

### 2.1. Proposed AI-Enabled Mobile System

**Figure 1:**
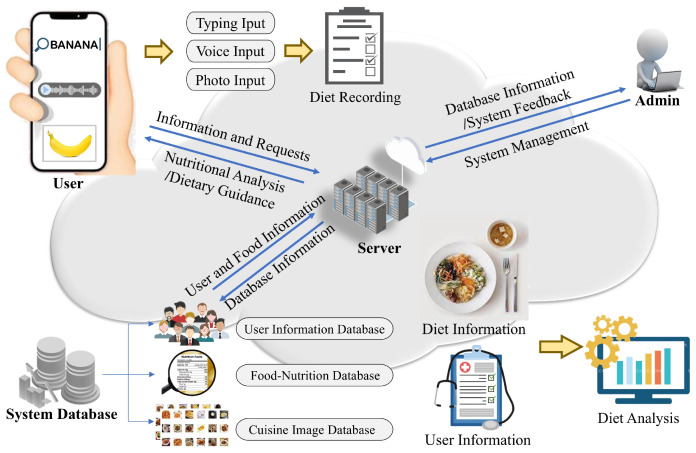
The framework of the AI-enabled mobile system.

The overall framework of the proposed AI-enabled system is illustrated in Figure 1. The system integrates typing, voice, and photo inputs for diet recording and evaluation. Its key components include a comprehensive system database, deep learning algorithms optimized for voice and diet-related image processing, and a smartphone application. Users interact with the system through a smartphone application, which allows them to manage personal and dietary information and receive detailed diet analysis and guidance.

#### 2.1.1. System Database

A well-established database provides critical data support and management to ensure efficient system functions and user input processing. In this mobile system, the system database consists of three main components: a user information database, a food–nutrition database, and a cuisine image database. The user information database stores basic information about the user, such as height and gender.

The food–nutrition database integrates both public and proprietary data sources. It contains nutritional information for over 1300 ingredients with more than 70 essential nutrients, sourced from the Chinese Food Composition [24,25]. This database also includes ingredient composition and nutritional data for over 5200 dishes, with the nutrition values calculated by the nutrients of their respective ingredients. Additionally, it comprises over 20,000 packaged foods, covering five core nutrients. The Chinese Food Composition is a publicly available reference, while the data for packaged foods and the ingredient composition of dishes were self-collected and compiled. These data are accessible for users to query and record, but personal dietary records from users remain private and are not shared. Table 1 illustrates the nutrient values per 100 g of ingredients and packaged foods, and the ingredient composition for dishes. This food–nutrition database enables the system to convert user records into detailed nutritional information for effective analysis.

Furthermore, we built a cuisine image database including nearly 5600 Chinese cuisine images to develop automatic food recognition and food weight estimation algorithms. This database is continuously expanding to enhance its comprehensiveness.

#### 2.1.2. User Information Management

The system records users’ physical, lifestyle habits, and medical information through manual entry, storing these data in the user information database that can be modified and updated as needed. Specifically, users complete a questionnaire via the application to capture essential characteristics such as name, gender, age, height, weight, exercise intensity, medical history, and special needs such as disease management or weight control. This information serves as the basis for personalized diet analysis and evaluation.

#### 2.1.3. Diet Recording Methods

Traditional diet evaluation systems require users to manually input food types, select corresponding items from a database, and enter the food’s weight. The proposed system enhances this process by offering more convenient voice and photo input methods, in addition to the conventional manual entry.

A. Diet Recording by Voice Input

**Figure 2:**
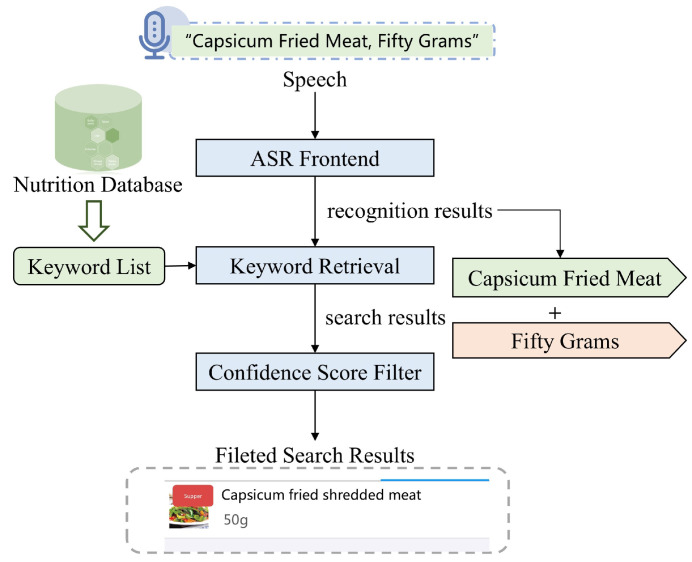
Schematic diagram of the voice-based dietary intake recording method.

The voice-based method employs the architecture of an efficient end-to-end ASR-KWS system [26], combining automatic speech recognition (ASR) with keyword search (KWS). In this method, as depicted in Figure 2, users input speech in the format “food name” and “food weight”, such as “capsicum fried meat, fifty grams”. The ASR decodes the input to extract phonemes of potential food names and weights. The system then matches the recognized food name with the food–nutrition database, using keywords to retrieve and filter results by timestamp and confidence provided by the alignment of grapheme and phoneme outputs. The final recorded result is “capsicum fried shredded meat, fifty grams”, matched with the database and presented in a visually rich format showing both text and image of the target dish.

B. Diet Recording by Single-Shot Cuisine Photography

**Figure 3:**
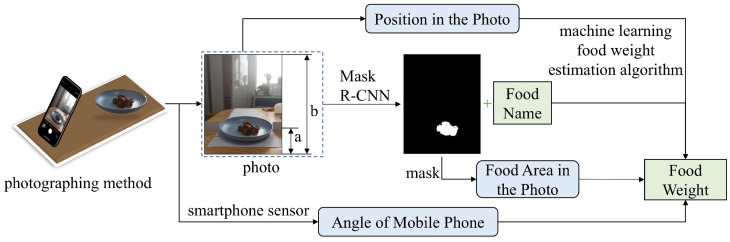
Schematic diagram of the single-shot-photography-based diet recording method for food identification and weight estimation.

Typing or voice input cannot automatically estimate food weight. Hence, we develop a machine vision-based diet recording method, as illustrated in Figure 3. The method can identify food and estimate weight through a single-shot cuisine photograph. It incorporates a deep learning model for food identification and a regression model for weight estimation using identified food types, photo-shooting angles, and photo-shooting distances. Existing methods that estimate food volume from image contours are subject to errors. In contrast, our model improves accuracy by considering the 3D-to-2D projection, calibrating the precision of volume estimation from 2D images using the shooting angle provided by the smartphone’s internal attitude sensors and distance calculated through perspective transformation. The single-shot diet recording process involves placing the smartphone on a table, adjusting the tilt for clear visibility, and capturing an image of the food. Then, a deep learning algorithm is utilized to obtain the name and food mask and determine the food’s projected area from the mask. The shooting angle and phone parameters (geometrical dimensions and built-in attitude sensors) are used to calculate the distance between the smartphone and the food. Finally, machine learning regression techniques combine these features to estimate the food weight.

**Figure 4:**
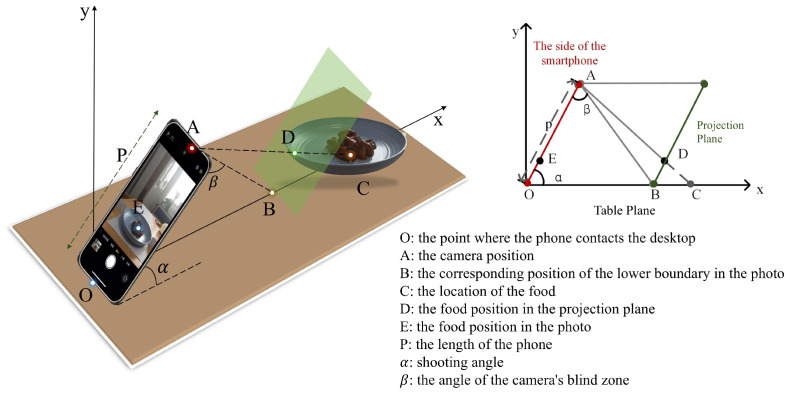
Illustration of the perspective transformation model for photo-shooting distance estimation.

We adopt the Mask R-CNN architecture [27] for food identification, which can recognize the food type and generate a food mask, allowing the extraction of the food’s projected area from the image. The distance between the smartphone and the target food is key to calibrating the actual food weight from 2D images. This photo-shooting distance can be estimated geometrically using a perspective transformation method. Figure 4 shows the side view of the photography scenario, with the phone–table contact point as the origin of the coordinate system. The *x*-axis and *y*-axis represent the directions along and vertical to the table, respectively. The phone’s length, denoted as *p*, corresponds to the distance from the bottom of the phone to the camera. The values of *p* and β depend on the phone model used, and the shooting angle α can be obtained from the phone’s gyroscope sensor. The calculation process is detailed as follows.

First, the coordinate of camera location *A* can be calculated by

(1) $$\left(x_{A},y_{A}\right)=\left(p\cos\alpha \;,\;p\sin\alpha \right),$$

and the length of OB is calculated according to β:

(2) $$l_{OB}=\frac{p\sin\beta }{\sin(\alpha +\beta )}$$.

Since the projected image is proportional to the actual image, the position of point *C* can be calculated by point *D*. The geometric parameters *a* and *b* represent the food’s position in the image, where *a* denotes the distance from the food to the bottom of the photo and *b* represents the total length of the image. As shown in Figure 4, the values of *a* and *b* can be obtained from the image. Let $k=\frac{a}{b}$, obtaining $\frac{l_{OE}}{p}=k$. Then, the coordinates of point *D* are calculated as follows:

(3) $$\left(x_{D},y_{D}\right)=\left(\frac{2pk\sin\alpha \cos\beta }{\sin(\alpha +\beta )}\;\cdot \;\cos\alpha \;,\;\frac{2pk\sin\alpha \cos\beta }{\sin(\alpha +\beta )}\;\cdot \;\sin\alpha \right).$$

Finally, the coordinate of point *C* can be calculated by giving points *D* and *A*. The shooting distance is given by

(4) $$l_{OC}=\frac{y_{D}\left(x_{D}-x_{A}\right)}{\left(y_{D}-x_{A}\right)}+x_{A}.$$

From the previous steps, we obtain the shooting tilt angle α, shooting distance $l_{OC}$, and food’s projected area *S* through masking from the image. These parameters serve as features to estimate food weight, formulating a multi-feature linear regression problem. To support this, we constructed a cuisine dataset of approximately 5600 samples of 19 foods and fruits, with cuisine photos taken from various angles and labeled with actual weights. Among the regression algorithms tested, the Least Absolute Shrinkage and Selection Operator (LASSO) showed the best performance [28], with a mean absolute error (MAE) of about 9.57 g. Compared to methods relying solely on 2D projected area, our approach improves accuracy by incorporating 3D parameters while requiring less sophisticated equipment than 3D modeling-based methods. The cuisine dataset is expanding to include more food types and shooting conditions to support dietary recording more effectively.

### 2.2. The Smartphone Application for Data Recording and Analysis

**Figure 5:**
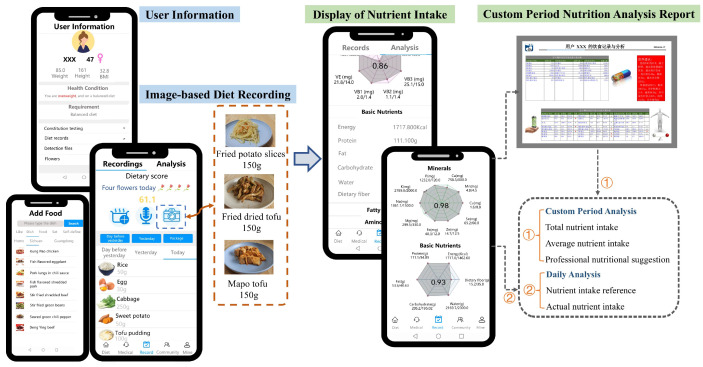
The implementation of the AI-enabled mobile system via smartphone application.

Figure 5 illustrates the smartphone application with functional modules for managing personal information, recording daily diets, and accessing nutritional analysis. The system determines nutrient intake reference values based on the user’s gender, age, height, weight, daily activity level, and other relevant data by the Dietary Reference Intakes For China [29]. These values can be adjusted according to medical advice. The recorded food names and weights are matched with the food-nutrition database to calculate actual nutrient intake. Finally, the system generates dietary analysis reports and nutritional radar charts to help users understand and improve their eating habits. Additionally, the system offers a communication channel with professional nutritionists for personalized guidance and provides educational courses on diet and health. It also integrates with external devices like bracelets and body fat scales to monitor physical status, including weight, body fat, heart rate, sleep, and stress levels.

### 2.3. Diabetes Progression Analysis

HbA1c dynamics serve as a crucial indicator of diabetes progression. This study proposed a comprehensive approach for analyzing HbA1c dynamics using multivariate data, focusing on diabetes-associated risk factors.

#### 2.3.1. DiabMini Dataset

To support this analysis, we introduce the DiabMini dataset, collected from a 3-week type 2 diabetes research project with Tianjin Third Central Hospital through the application.

The data collection process was designed with domain experts and had two main components. The first was gathering participant data through the application, including personal information, lifestyle, nutrient intake, and medical examination data [30]. The second component involved working with professional physicians and nutrition specialists to filter and annotate the integrated data. DiabMini comprises 88 samples, each characterized by 127 features across four categories.

1. Personal information (*n* = 6): Including basic information such as height and weight;
2. Medical information (*n* = 32): Participants underwent two medical examinations, one before and one after the project, recording 16 test indicators through routine blood tests and body composition analysis. Both examinations were conducted by professional hospitals using consistent instruments and procedures;
3. Lifestyle (*n* = 10): Lifestyle data were collected through an online questionnaire in the application. It contained ten questions about activities and habits, such as exercise frequency and sleep duration. These questions were designed based on physician expertise and diabetes risk factors;
4. Nutrient intake (*n* = 79): Between two medical examinations, all participants were required to record their complete daily dietary intake for 14 days, including meals, beverages, snacks, and fruits. These records were automatically converted into the intake of 79 specific nutrients. The convenient recording methods and extensive food-nutrient database of the application ensured efficient dietary tracking.
  The detailed annotation of all data is presented in Appendix A.

#### 2.3.2. Model Establishment and Evaluation

All 88 participants in the DiabMini dataset had two medical examinations that included HbA1c indicators. We categorized the HbA1c changes as follows: 0 = No change, 1 = Increased, and 2 = Decreased, based on a comparison between the second and first measurements. After data processing, the number of samples of Class 0, Class 1, and Class 2 is 21:15:52. Using these HbA1c changes as the target variable, and 6 personal, 10 lifestyle, 79 nutrient intake, and 16 initial medical features as predictors, we conducted a three-class prediction experiment. The average nutrient intake of each participant was used to balance short-term fluctuations and capture long-term dietary habits.

For HbA1c prediction, we employed a stacking model architecture. Rather than relying on a single machine learning algorithm, model stacking combines the strengths of multiple algorithms, mitigating overfitting and enhancing overall predictive performance and generalization. Considering the small sample size and high feature dimensionality of DiabMini, we selected XGBoost, SVC, and ET as base models, with KNN as the meta-model. XGBoost is well-suited for handling imbalanced data distributions, SVC efficiently addresses complex classification tasks, and ET offers robustness against noise. These models perform well in high-dimensional spaces, making them ideal for datasets with limited samples but many features. KNN was chosen as the meta-model for its capability in multi-class classification and its suitability for small datasets. Furthermore, they can be seamlessly integrated with model interpretation methods for model output visualization.

We assessed model performance using StratifiedKFold cross-validation. This method randomly divides the dataset into multiple folds in each iteration while ensuring that the class distribution within each fold is consistent with the original dataset, thereby minimizing performance evaluation bias caused by uneven data splitting. Additionally, we applied the SMOTE oversampling technique to interpolate and synthesize new samples from the original minority class. It is commonly used in the healthcare domain to address class imbalance.

Common evaluation metrics for multi-class classification tasks include accuracy and the macro-averages of precision, recall, and F1-score, which we used for the HbA1c classification problem. The respective definitions of *TP*, *FP*, *TN*, and *FN* are true positive, false positive, true negative, and false negative. The multi-classification metrics are defined as follows:

(5) $$\begin{array}{cc} & \mathrm{Accuracy}=\frac{\sum_{i=1}^{n}\;TP_{i}}{\sum_{i=1}^{n}\;\left(TP_{i}+FN_{i}\right)}, \end{array}$$

(6) $$\;\mathrm{Macro-Precision}\;=\frac{1}{n}\sum_{i=1}^{n}\;\frac{TP_{i}}{TP_{i}+FP_{i}},$$

(7) $$\;\mathrm{Macro-Recall}=\frac{1}{n}\sum_{i=1}^{n}\;\frac{TP_{i}}{TP_{i}+FN_{i}},$$

(8) $$\;\mathrm{Macro-F1}\;=2\times \frac{\mathrm{Macro-Precision}\times \mathrm{Macro-Recall}}{\mathrm{Macro-Precision}+\mathrm{Macro-Recall}},$$

where *n* is the total number of classes, and *i* denotes the *i*-th class (i=1,2,⋯n). In multi-class scenarios, $\sum_{i=1}^{n}\left(TP_{i}+FN_{i}\right)=\sum_{i=1}^{n}\left(TP_{i}+FP_{i}\right)=\mathrm{Total}\;\mathrm{samples}$.

Additionally, we utilized the ROC curve and confusion matrix for model performance visualization. The ROC curve illustrates the model’s performance across various decision thresholds, while the confusion matrix provides a detailed view of the classification outcomes.

## 3. Results

### 3.1. HbA1c Classification Experimental Results

The results of the stacking model for HbA1c classification are listed in Table 2, which presents a comprehensive comparison of model performance across various configurations of base and meta models. The configuration that utilizes all three base models (XGBoost, SVC, and ET) in combination with KNN as the meta-model achieves the best performance, with an accuracy of 94.23%, macro-precision of 94.97%, macro-recall of 94.24%, and macro-F1 of 94.16%. In contrast, using KNN alone as the meta-model without any base models results in the lowest performance metrics, particularly with an accuracy of 73.04% and a macro-F1 score of 68.64%. These findings suggest that the proposed stacking model, which combines multiple base models with a robust KNN meta-model, is highly suitable for the structure of the DiabMini dataset. It effectively addresses the challenges posed by the high feature dimensionality and small sample size of the DiabMini in classification tasks. By leveraging the complementary strengths of various algorithms, the stacking approach improves classification accuracy and the model’s generalization across different classes, making it an ideal solution for our specific classification problem.

**Figure 6:**
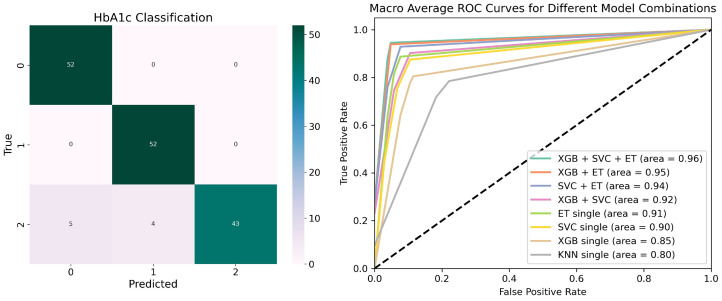
The confusion matrix and ROC curve for HbA1c classification model performance.

Figure 6 depicts the confusion matrix (left) and ROC curve (right), illustrating the performance of the HbA1c classification model in detail.

The confusion matrix shows the performance by comparing true class labels (vertical axis) with the predicted labels (horizontal axis), where diagonal elements represent correctly classified instances for each class. The color intensity reflects the number of instances, with darker representing higher counts of correctly or incorrectly classified instances. As observed, the model accurately classified the majority of instances across all three classes, particularly in Class 0 (No change) and Class 1 (Increased). Several misclassifications occurred in Class 2 (Decreased), where a few samples were incorrectly predicted as Class 0 or Class 1. This misclassification likely stems from the inherent imbalance and overlapping feature spaces between the classes. Despite applying SMOTE to address class imbalance, the complexity of the dataset remains a challenge commonly encountered in real-world applications. Despite these challenges, the confusion matrix indicates that the model performs well in predicting HbA1c changes. More real-world samples will be collected in the future to mitigate class imbalance and improve performance across all classes.

The ROC curve graph is a probability curve reflecting the relationship between true positive rate (TPR) and false positive rate (FPR). The Area Under Curve (AUC) is the area enclosed by the ROC curve and the x-axis, which is used to quantify the classification ability of the model. The value of AUC ranges from 0.5 to 1.0, with a higher AUC indicating better performance. Figure 6 displays the macro average ROC curves for different model combinations in the HbA1c classification task, with KNN serving as the meta-model. The ensemble of XGB, SVC, ET, and KNN achieves the highest AUC of 0.96, showing superior classification performance. The second-best combination, XGB + ET, reaches an AUC of 0.95, while SVC + ET achieves 0.94. Among the single models, ET performs well with an AUC of 0.91, followed by SVC (0.90) and XGB (0.85). KNN shows a relatively lower AUC of 0.80. These results demonstrate that ensemble models generally outperform single models in this classification task, with the four-model combination ensemble delivering the best results.

### 3.2. HbA1c Classification Model Interpretability

Beyond the robust and reliable model performance, model interpretability is imperative, especially in the healthcare domain where critical decisions are made. Model interpretability enables clinicians and patients to comprehend the working principle and output of the model, facilitating reasonable adjustments for personalized health management. Recently, the SHAP has gained widespread adoption for interpreting machine learning models [31]. It calculates the SHAP value by considering the average marginal contribution of each feature across all possible features to assess their attribution on the model prediction. A higher SHAP value indicates the feature’s greater influence over the model output.

**Figure 7:**
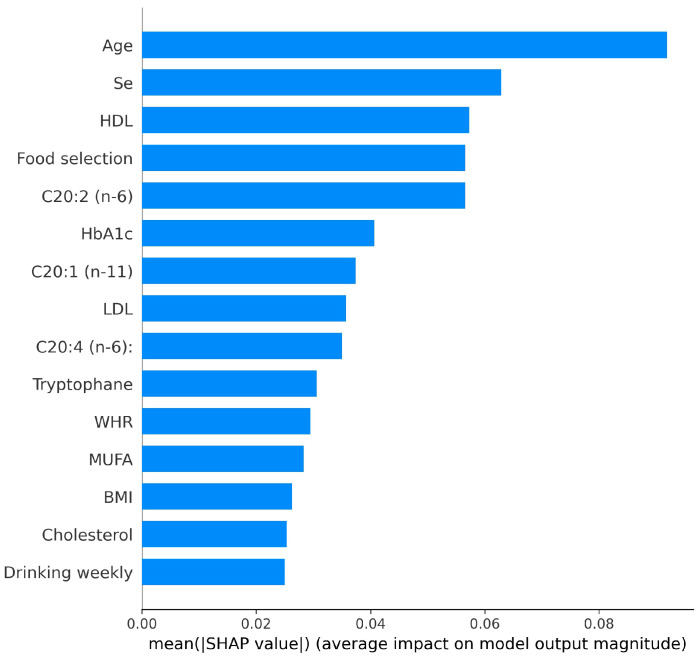
Top 15 global features ranked by SHAP for HbA1c prediction.

Figure 7 displays the SHAP summary plot of global feature importance for the HbA1c prediction model using the ensemble of XGB, SVC, ET, and KNN. The X-axis represents the mean SHAP values of the feature across all samples, indicating the magnitude of each feature’s contribution to the model’s predictions. The Y-axis is the descending order according to this value. As shown in the figure, the feature with the greatest impact on the overall classification is age, followed by selenium (Se) and high-density lipoprotein (HDL) in the first medical examination. These variables have the highest SHAP values, reflecting their strong impact on the model’s output. Other important features include food selection, fatty acids cis, cis-11,14-Eicosadienoic (C20:2 (n-6)) and cis-9-Eicosenoic (C20:1 (n-11)), and HbA1c from the first medical examination. Features such as low-density lipoprotein (LDL), tryptophan, and waist-to-hip ratio (WHR) also contribute significantly. This plot emphasizes the need to consider multiple factors throughout the prediction process, highlighting that nutrient intake, lifestyle factors, and clinical biomarkers are all indispensable in HbA1c dynamic prediction.

**Figure 8:**
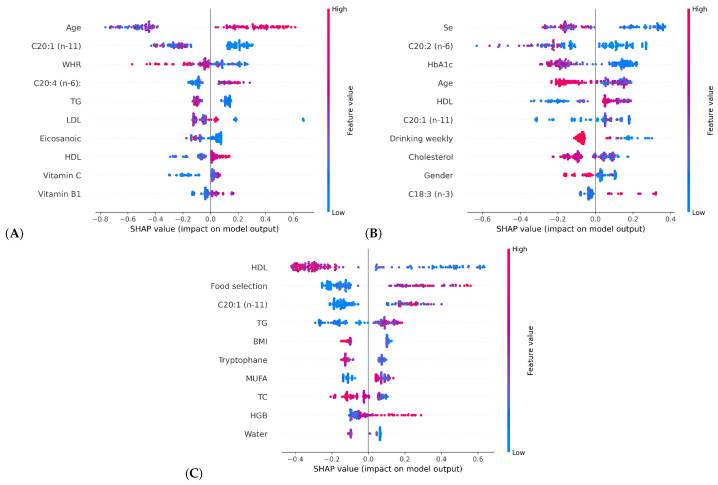
Top 10 local features ranked by SHAP for HbA1c prediction of three classes. (**A**) is Class 0 (no change), (**B**) denotes Class 1 (increased), and (**C**) is Class 2 (decreased).

In particular, for each class, the importance of features and their impact on the output are presented in Figure 8. The Y-axis is the features ranked by their importance, while the X-axis is the SHAP value of the features. Positive and negative SHAP values indicate the positive and negative influences of the features on the predictions, respectively. Each point in the figure represents a sample, with red representing high feature values and blue indicating low feature values.

For Class 0, age, C20:1 (n-11), and WHR are the most influential features. Age has the largest positive SHAP values, indicating that older age has a greater impact on the model output for this class. C20:1 (n-11) ranks second, with its distribution showing that lower levels of this fatty acid have a small impact on HbA1c dynamics [32]. The third feature is WHR, where the concentration of blue dots on the right side of the X-axis suggests that lower WHR tends to help maintain HbA1c stability.

For Class 1, Se, C20:2 (n-6), and HbA1c play important roles. The Se shows a strong positive correlation with the model output, indicating that lower Se intake may lead to increased HbA1c. This observation aligns with research suggesting that appropriate Se intake can improve diabetes condition [33,34]. The C20:2 (n-6) has a similar distribution to Se, indicating that sufficient intake of it may also contribute to better diabetes control [35].

In Class 1 and Class 2, HbA1c and HDL from the first medical examination show more significance. Their distributions reveal that lower initial HbA1c and higher HDL are associated with future increases in HbA1c, contradicting common perceptions [36,37]. This phenomenon relates to “diabetes burnout” reported in recent studies [38]. Specifically, low HbA1c and high HDL at the first examination imply well-controlled current glycemic and may lead to patients’ laxity in personal diabetes management, resulting in deteriorating glycemic status in the future. Conversely, high HbA1c and low HDL at the first examination can alert patients and prompt them to better blood glucose control. A similar pattern is observed with serum triglycerides (TG) in Class 0 and 2. Lower TG from the first medical examination tends to stabilize future HbA1c levels, whereas higher TG levels are associated with a decrease in future HbA1c. This finding is consistent with the studies that highlight TG as a key predictive factor for diabetes, due to its strong elevation with the exacerbation of diabetes [39,40,41]. As a result, high TG in the initial examination may encourage patients to take stricter measures in managing blood glucose.

The plots for Class 1 and Class 2 also emphasize the importance of lifestyle habits. The concentration of red points on the left side of the SHAP values for drinking weekly indicates that, when the frequency is “Never or rarely” (with 69 samples), its impact tends to reduce HbA1c. The points distribution for food selection indicates that choosing foods based on individual health conditions rather than solely on personal preference or nutritional value is more conducive to controlling HbA1c.

## 4. Discussion and Conclusions

Monitoring diabetes progression is one of the core tasks in diabetes management. Current methods mainly rely on patient self-reporting, which is prone to inaccuracies, misreporting, and inconsistent data collection. Furthermore, existing data analysis approaches often lack the comprehensive integration of data from multiple sources. This study developed an innovative solution combining an AI-enabled mobile system for extensive data collection with appropriate machine learning techniques to predict HbA1c dynamics for better diabetes management.

The AI-enabled mobile system efficiently and continuously collects various user data, leveraging deep learning technologies and an extensive system database to improve convenience and accuracy. By capturing a wide range of user data, from basic characteristics such as height and weight to more specific details like nutrient intake and lifestyle habits, the system provides a robust foundation for diabetes progression analysis. Building on this data collection capability, we developed the DiabMini dataset to analyze the factors influencing diabetes progression. Since HbA1c dynamics reflect changes in diabetes progression, we applied machine learning to explore how personal, medical, nutrient intake, and lifestyle factors affect HbA1c dynamics. The predictive accuracy of the stacking model demonstrates the value of incorporating comprehensive variables. SHAP analysis further enhanced the interpretability and transparency of the model by clearly visualizing the relationships between these variables and HbA1c outcomes.

This approach effectively predicts diabetes progression and ensures the collection of necessary data conveniently via the proposed mobile system. Unlike recent research that primarily focused on clinical data such as CGM, medical examination, personal information, or electronic health record [15,16,17], our approach integrates nutrient intake and lifestyle factors, which are often overlooked but crucial in influencing HbA1c dynamics. Key dietary and lifestyle elements, such as Se intake and frequency of drinking, were identified as significant predictors, underscoring the importance of considering nutrition and lifestyle in diabetes management.

In conclusion, this study demonstrates the potential of integrating the smartphone application with machine learning for diabetes management. The combination of comprehensive data collection, thorough data analysis, and accurate predictions offers a practical tool for improving diabetes care. Its ease of implementation can reduce the financial and time burdens for long-term diabetes monitoring. Future research will aim to expand the dataset and validate the system and model in larger, real-world clinical environments, enhancing both predictive capabilities and practical utility in diabetes management.

## Figures and Tables

**Table 1:** Sample information from the food–nutrition database.

Ingredient	Energy	Fat	Carbohydrate	Protein	Water	Dietary Fiber	Vitamin B1	Ca	Fe	Na	...
Rice	116 kcal	0.3 g	25.9 g	2.6 g	70.9 g	0.3 g	0.02 mg	7 mg	1.3 mg	2.5 mg	
Potato	77 kcal	0.2 g	17.2 g	2 g	79.8 g	0.7 g	0.08 mg	8 mg	0.8 mg	2.7 mg	
Pork	395 kcal	37 g	2.4 g	13.2 g	46.8 g	- ^*^	0.22 mg	6 mg	1.6 mg	59.4 mg	
Chicken	167 kcal	9.4 g	1.3 g	19.3 g	69 g	- ^*^	0.05 mg	9 mg	1.4 mg	63.3 mg	
Mushroom	24 kcal	0.1 g	4.1 g	2.7 g	92.4 g	2.1 g	0.08 mg	6 mg	1.2 mg	8.3 mg	
Tomato	20 kcal	0.2 g	4 g	0.9 g	94.4 g	0.5 g	0.03 mg	10 mg	0.4 mg	5 mg	
...											
**Packaged Foods**	**Energy**	**Fat**	**Carbohydrate**	**Protein**	**Na**						
Oatmeal	377 kcal	6.7 g	66.9 g	15 g	3.7 mg						
Biscuit	435 kcal	12.7 g	71.7 g	9 g	204.1 mg						
Fried Chips	615 kcal	48.4 g	41.9 g	4 g	60.9 mg						
Spicy Kelp	57.9 kcal	1.7 g	8.5 g	3.1 g	2590 mg						
Cheese	328 kcal	23.5 g	3.5 g	25.7 g	584 mg						
...											
**Dishes**	**Ingredient**	**Amount**	**Ingredient**	**Amount**	**Ingredient**	**Amount**	**Ingredient**	**Amount**	**Ingredient**	**Amount**	**...**
Spaghetti with Sauce	Macaroni	300 g	Pork	100 g	Tomatoes	100 g	Onion	50 g	Pepper	3 g	
Sandwich	Bread	100 g	Luncheon Meat	80 g	Cucumber	50 g	Tomato	50 g	Lettuce	30 g	
Yam Sparerib Porridge	Yam	150 g	Pork Chop	150 g	Rice	150 g	Water	400 g	Coriander	10 g	
Meat Floss Sushi	Rice	200 g	Pork Floss	50 g	Vinegar	5 g	Laver	3 g	Cucumber	30 g	
Roast Chicken	Rice	250 g	Chicken	200 g	Cucumber	80 g	Chinese Onion	10 g	Ginger	10 g	
...											

^*^—indicates the absence of this nutrient.

**Table 2:** Performance of HbA1c classification models.

Base Model			Meta Model	Evaluation Metrics (%)			
**XGBoost**	**SVC**	**ET**	**KNN**	**Accuracy**	**Macro-Precision**	**Macro-Recall**	**Macro-F1**
✓	-	-	-	80.18 ± 6.66	82.70 ± 4.68	80.42 ± 6.33	79.91 ± 7.09
-	✓	-	-	86.51 ± 3.24	87.69 ± 3.67	86.55 ± 3.58	85.69 ± 3.82
-	-	✓	-	88.45 ± 3.33	89.40 ± 2.98	88.42 ± 3.42	88.46 ± 3.24
-	-	-	✓	73.04 ± 2.83	80.29 ± 2.10	73.15 ± 3.05	68.64 ± 3.95
✓	✓	-	✓	89.11 ± 2.52	91.16 ± 1.84	89.15 ± 2.73	88.59 ± 2.92
✓	-	✓	✓	93.59 ± 2.04	94.29 ± 1.76	93.64 ± 1.84	93.42 ± 2.08
-	✓	✓	✓	92.34 ± 3.16	93.36 ± 2.65	92.48 ± 2.94	92.12 ± 3.40
✓	✓	✓	✓	94.23 ± 1.27	94.97 ± 1.07	94.24 ± 1.24	94.16 ± 1.26

The ✓ indicates that the algorithm is used, while a - indicates it is not.

## Data Availability

The data that support this study are available from the link: https://github.com/JiangHY616/DiabMini-Dataset, accessed on 16 October 2024.

## Appendix A

The complete features in DiabMini used for diabetes analysis.

Personal Information (*n* = 6):

- Age— age, years
- Gender—(1—Female, 2—male)
- Chronic disease—(1—Heart disease, 2—Diabetes, 3—No, 4—Hypertension)
- Height—height, centimeter
- Weight—weight, kilogram
- BMI—Body Mass Index

Medical Information (*n* = 32): There are two examinations in total, and one medical examination contains 16 examination indicators.

- BFP: Body fat percentage, the proportion of total body fat to body weight
- WHR: Waist to hip ratio, the ratio of waist circumference to hip circumference
- VFA: Visceral fat area, refers to the tomographic area index of visceral fat in CT imaging
- HbA1c: Glycosylated hemoglobin, the compounds that bind glucose and hemoglobin
- FBG: Fasting blood glucose, the blood glucose measured before breakfast the next morning for more than eight to twelve hours of fasting overnight
- TP: Total protein, the general name of albumin and globulin
- Alb: Albumin, the most important protein in human plasma
- TG: Serum triglycerides, the important component of blood lipids, mmol/L
- TC: Serum total cholesterol, the sum of cholesterol contained in all lipoproteins in the blood, mmol/L
- HDL: High-density lipoprotein, one of the serum proteins
- LDL: Low-density lipoprotein, one of the lipoprotein components in blood lipids
- AI: Arteriosclerosis index, the index to evaluate the degree of arteriosclerosis
- WBC: White blood cell, the cells with motility and phagocytosis
- RBC: Red blood cells, the most numerous types of blood cells in the blood
- HGB: Hemoglobin, the protein contained in red blood cells
- LY: Lymphocyte count, to count lymphocytes and calculate the percentage

Lifestyle (*n* = 10):

- Working hours—(1—8–12 h, 2—4–8 h, 3—Less than 4 h, 4— More than 12 h)
- Work intensity—(1—Student, 2—Office work, 3—Retire, 4—High intensity)
- Exercise weekly—(1—3–4 times, 2—1–2 times, 3—Occasionally or hardly, 4—More than 5 times)
- Sleep time—(1—4–6 h, 2—6–8 h, 3—More than 8 h, 4—Less than 4 h)
- Meal habits—(1—No breakfast, 2—Very irregular, 3—Three meals are regular)
- Food selection—(1—Pay attention to nutritional value, 2—Personal preference, 3—Careful selection based on health condition)
- Dietary preferences—(1—Vegetarian diet, 2—Balanced diet, 3—Meat diet)
- Water intake daily—(1—1000–2000 mL, 2—2000–3000 mL, 3—More than 3000 mL, 4—0–1000 mL)
- Drinking weekly—(1—3–4 times, 2—Less than 3 times, 3—Never or rarely, 4—More than 5 times)
- Smoking daily—(1—5–10 cigarettes, 2—1–5 cigarettes, 3—No smoking, 4—More than 10 cigarettes)

Dietary Content (*n* = 79):

- Cholesterol: the component of lipids, mg
- Purine: the organic compounds produced by the body’s metabolism, mg
- Energy: kilocalorie, kcal
- Protein: g
- Fat: g
- Carbohydrate: g
- Water: g
- Dietary fiber: g
- Total vitamin A: μg
- Vitamin E: mg
- Vitamin B1: mg
- Vitamin B2: mg
- Vitamin B3: mg
- Vitamin C: mg
- Ca: Calcium, mg
- P: Phosphorus, mg
- K: Potassium, mg
- Na: Sodium, mg
- Mg: Magnesium, mg
- Fe: Iron, mg
- Zn: Zinc, mg
- Se: Selenium, μg
- Cu: Copper, mg
- Mn: Manganese, mg
- Total fatty acids: g
- SFA: Saturated fatty acids, g
- MUFA: Monounsaturated fatty acid, g
- PUFA: Polyunsaturated fatty acid, g
- Hexanoic (Caproic), mg
- Octanoic (Caprylic), mg
- Decanoic (Capric), mg
- Henedecanoic (Undecylic), mg
- Dodecanoic (Lauric), mg
- Tridecanoic (Tridecylic), mg
- Tetradecanoic (Myristic), mg
- Pentadecanoic (Pentadecylic), mg
- Hexadecanoic (Palmitic), mg
- Heptadecanoic (Margaric), mg
- Nonadecanoic (Nondecylic), mg
- Eicosanoic (Arachidic), mg
- Docosanoic (Behenic), mg
- C14:1 (n-5): cis-9-Tetradecenoic, Myristoleic, mg
- C15:1 (n-5): 10-Pentadecenoic, mg
- C16:1 (n-7): cis-9-Hexadecenoic, Palmitoleic, mg
- C17:1 (n-7): 10-Heptadecenoic, mg
- C18:1 (n-9): cis-9-Octadecenoic, Oleic, mg
- C20:1 (n-11): cis-9-Eicosenoic, Gadoleic, mg
- C22:1 (n-13): cis-9-Docosenoic, mg
- C16:2 (n-4): cis, cis-9,12-Hexadecadienoic, mg
- C18:2 (n-6): cis, cis-9,12-Octadecadienoic, Linoleic, mg
- C18:3 (n-3): all cis-9,12,15-Octadecatrienoic, α-Linolenic, mg
- C20:2 (n-6): cis, cis-11,14-Eicosadienoic, mg
- C20:3 (n-9): all cis-5,8,11-Eicosatrienoic, Mead, mg
- C20:4 (n-6): all cis-5,8,11,14-Eicosatetraenoic, Arachidonic, mg
- C20:5 (n-3): all cis-5,8,11,14,17-Eicosapentaenoic, mg
- C22:3 (n-3): all cis-13,16,19-Docosatrienoic, mg
- C22:4 (n-6): all cis-7,10,13,16-Docosatetraenoic, mg
- C22:5 (n-3): all cis-7,10,13,16,19-Docosapentaenoic, mg
- C22:6 (n-3): all cis-4,7,10,13,16,19-Docosahexaenoic, mg
- Isoleucine, mg
- Leucine, mg
- Lysine, mg
- TSAA: Total sulfur containing amino acids, mg
- Methionine, mg
- Cystine, mg
- TAAA: Total aromatic amino acids, mg
- Phenylalanine, mg
- Tyrosine, mg
- Threonine, mg
- Tryptophane, mg
- Valine, mg
- Arginine, mg
- Histidine, mg
- Alanine, mg
- Aspartic acid, mg
- Glutamic acid, mg
- Glycine, mg
- Proline, mg
- Serine, mg
